# Supplementary material for: Mechanisms governing the pioneering and redistribution capabilities of the non-classical pioneer PU.1
Source: Nat Commun. 2020 Jan 21;11:402. doi: 10.1038/s41467-019-13960-2 (PMC6972792; doi:10.1038/s41467-019-13960-2)
Supplement: Supplementary file 7 — Source data [file 41467_2019_13960_MOESM7_ESM.zip › Source_Data/Figure6/Figure6B_MotifScanOutput/ETS-specific/homerResults/motif36.similar.html]

motif36

## Information for motif36

A
G
T
C
A
G
T
C
C
T
G
A
T
A
G
C
G
A
C
T
C
T
G
A
T
A
C
G
T
C
G
A
A
C
T
G
T
C
A
G
T
A
C
G
G
T
A
C
T
C
A
G
A
T
G
C
  
Reverse Opposite:  

T
A
C
G
A
G
T
C
A
C
T
G
A
T
G
C
A
G
T
C
T
G
A
C
A
G
C
T
A
T
G
C
G
A
C
T
C
T
G
A
A
T
C
G
G
A
C
T
C
T
A
G
T
C
A
G
  

|  |  |
| --- | --- |
| p-value: | 1e-25 |
| log p-value: | -5.829e+01 |
| Information Content per bp: | 1.648 |
| Number of Target Sequences with motif | 125.0 |
| Percentage of Target Sequences with motif | 1.63% |
| Number of Background Sequences with motif | 178.4 |
| Percentage of Background Sequences with motif | 0.44% |
| Average Position of motif in Targets | 102.8 +/- 57.8bp |
| Average Position of motif in Background | 87.0 +/- 63.8bp |
| Strand Bias (log2 ratio + to - strand density) | 0.1 |
| Multiplicity (# of sites on avg that occur together) | 1.02 |
| Motif File: | file (matrix) reverse opposite |

### Similar de novo motifs found

|  |  |  |  |  |  |  |  |
| --- | --- | --- | --- | --- | --- | --- | --- |
| Rank | Match Score | Redundant Motif | P-value | log P-value | % of Targets | % of Background | Motif file |
| 1 | 0.909 | G A T C A C T G A G T C G A T C G T A C A G C T A T G C G A C T T C G A T C A G A G T C A T C G C T A G | 1e-24 | -56.268883 | 1.10% | 0.21% | motif file (matrix) |
| 2 | 0.915 | T A G C T C G A A T G C G A C T T C G A A T C G C T G A C A T G T C A G T C A G G A T C C T A G | 1e-21 | -48.555375 | 1.76% | 0.59% | motif file (matrix) |
